# Zero Setup Margin Mask versus Frame Immobilization during Gamma Knife^®^ Icon™ Stereotactic Radiosurgery for Brain Metastases

**DOI:** 10.3390/cancers14143392

**Published:** 2022-07-13

**Authors:** Tugce Kutuk, Rupesh Kotecha, Ranjini Tolakanahalli, D Jay J. Wieczorek, Yongsook C. Lee, Manmeet S. Ahluwalia, Matthew D. Hall, Michael W. McDermott, Haley Appel, Alonso N. Gutierrez, Minesh P. Mehta, Martin C. Tom

**Affiliations:** 1 Department of Radiation Oncology, Miami Cancer Institute, Baptist Health South Florida, Miami, FL 33176, USA; tugcek@baptisthealth.net (T.K.); rupeshk@baptisthealth.net (R.K.); ranjinit@baptisthealth.net (R.T.); djaywi@baptisthealth.net (D.J.J.W.); yongsook.lee@baptisthealth.net (Y.C.L.); matthewha@baptisthealth.net (M.D.H.); haleya@baptisthealth.net (H.A.); alonsog@baptisthealth.net (A.N.G.); mineshm@baptisthealth.net (M.P.M.); 2 Department of Radiation Oncology, Herbert Wertheim College of Medicine, Florida International University, Miami, FL 33199, USA; 3 Department of Medical Oncology, Miami Cancer Institute, Baptist Health South Florida, Miami, FL 33176, USA; manmeeta@baptisthealth.net; 4 Department of Neurosurgery, Miami Cancer Institute, Baptist Health South Florida, Miami, FL 33176, USA; mwmcd@baptisthealth.net

**Keywords:** brain metastases, radiosurgery, SRS, mask, frameless, frame, zero setup margin

## Abstract

**Simple Summary:**

The Gamma Knife^®^ Icon™ allows for mask immobilization for stereotactic radiosurgery (SRS) as an alternative to frame immobilization. However, standardized recommendations for setup margins (SM) to create the planning target volume (PTV) with the mask immobilization do not exist and, therefore, practice patterns vary. Adding a SM might be the correct approach, if the possibility of significant intrafraction motion exists; on the other hand, it may be unnecessary as it increases the risk of radiation necrosis. This study, comprising 150 patients with 453 brain metastases (BM) treated for a median of 15 months of follow up, demonstrates that zero-SM mask immobilization had comparable clinical outcomes compared to a control group of similar patients undergoing frame immobilization SRS. There was no difference in freedom from local failure (FFLF) between the mask and frame immobilization groups on univariable or multivariable analysis. The initial findings support omitting a SM when using mask immobilization with this treatment approach on a GK Icon™.

**Abstract:**

We compared the clinical outcomes of BM treated with mask immobilization with zero-SM (i.e., zero-PTV) to standard zero-SM frame immobilization SRS. Consecutive patients with BM, 0.5–2.0 cm in maximal diameter, treated with single-fraction SRS (22–24 Gy) during March 2019–February 2021 were included. Univariable and multivariable analysis were performed using the Kaplan–Meier method and Cox proportional hazards regression. A total of 150 patients with 453 BM met inclusion criteria. A total of 129 (28.5%) lesions were treated with a zero-SM mask immobilization and 324 (71.5%) with zero-SM frame immobilization. Frame immobilization treatments were associated with a higher proportion of gastrointestinal and fewer breast-cancer metastases (*p* = 0.024), and a higher number of treated lesions per SRS course (median 7 vs. 3; *p* < 0.001). With a median follow up of 15 months, there was no difference in FFLF between the mask and frame immobilization groups on univariable (*p* = 0.29) or multivariable analysis (*p* = 0.518). Actuarial FFLF at 1 year was 90.5% for mask and 92% for frame immobilization (*p* = 0.272). Radiation necrosis rates at 1 year were 12.5% for mask and 4.1% for frame immobilization (*p* = 0.502). For BM 0.5–2.0 cm in maximal diameter treated with single-fraction SRS using 22–24 Gy, mask immobilization with zero SM produces comparable clinical outcomes to frame immobilization. The initial findings support omitting a SM when using mask immobilization with this treatment approach on a Gamma Knife^®^ Icon™.

## 1. Introduction

Brain metastases (BM) represent the most common malignancy in the central nervous system, which develop in approximately 30% of cancer patients [1]. Prospective randomized trials have established stereotactic radiosurgery (SRS) as the preferred method of treating BM among well-selected patients [2,3,4,5,6,7]. While multiple SRS platforms exist, the Gamma Knife^®^ (GK; Elekta, Stockholm, Sweden) was one of the first dedicated units specifically developed to perform intracranial SRS [8]. With GK, a head frame is attached to the cranium under local anesthesia in order to rigidly immobilize the head during treatment. With frame immobilization, no setup margin (SM) is required beyond the gross tumor volume (GTV) to create the planning tumor volume (PTV), thus limiting the volume of normal brain receiving the prescription dose of radiation [9]. Indeed, frame immobilization has been used for decades and allows for extended treatment durations in a single session, such as for patients with a higher number of BM. The potential disadvantages of using an invasive frame include discomfort with pin placement, the need to account for prior cranial surgeries during pin placement, rare cases of bleeding or infection, additional required monitoring with the administration of conscious sedation, and rare instances of frame slippage [10].

The latest model of the GK, the Icon^TM^, allows the use of a thermoplastic mask for immobilization [11]. Mask fixation represents a noninvasive form of immobilization, obviating the need for invasive frame placement and conscious sedation in select patients, while also allowing for multi-session treatment courses that are more commonly being used for larger target volumes [12]. Mask-based immobilization with a combination of on-board cone-beam computed tomography (CBCT) imaging, automatic co-registration, online rapid adaptive re-planning, and monitoring of the patient with continuous infrared guided high-definition motion management (HDMM) allows for submillimeter positional accuracy [13,14,15,16]. However, there are concerns that mask immobilization, even with HDMM, may allow for excess motion in comparison to frame immobilization [17,18,19,20]. When the rigid immobilization frame is dispensed with, clinical practice regarding SM varies by institutional preference, with some centers applying no margin [21,22] and others up to 1 mm, and in rare instances, even 2 mm [13,19,21,23]. However, the optimal SM required with mask immobilization and HDMM, if any, is unclear. Adding a SM might be the correct approach, if the possibility of significant intrafraction motion exists; on the other hand, it may be unnecessary as it increases the volume of uninvolved brain receiving radiation, and consequently the risk of radiation necrosis [24]. The primary objective of this study is to compare the clinical outcomes of BM treated using zero-SM during mask immobilization to a control group of zero-SM frame immobilization SRS on the GK Icon^TM^.

## 2. Materials and Methods

### 2.1. Data Acquisition

This study was approved by the institutional review board. We included consecutive BM patients with lesions 0.5–2.0 cm in maximal diameter treated with single-fraction SRS on the GK Icon^TM^ to a prescription dose of 22–24 Gy at a single tertiary care institution from February 2019 to January 2021. Lesions < 0.5 cm in maximal diameter were excluded given institutional practice at the time to preferentially treat these patients with frame immobilization. Similarly, lesions > 2.0 cm in maximal diameter were excluded due to institutional practice to treat them with hypofractionated or staged radiosurgery, given evidence suggesting poor local control (LC) with single-fraction SRS [25]. Patients treated with whole brain radiation therapy (WBRT) before SRS were excluded. Relevant patient data collected from the electronic medical records included sex, age, tumor histology, race, burden and status of extracranial disease, and Karnofsky Performance Status (KPS) at the time of SRS. Radiotherapy information (dose and fractionation, number of lesions treated, maximum lesion diameter, and lesion volume) was extracted from the Leksell GammaPlan^®^ treatment planning system (Elekta AB, Stockholm, Sweden).

### 2.2. Target Delineation

For patients undergoing SRS, dedicated treatment planning magnetic resonance images (MRIs) were obtained within 48 h preceding the delivery of treatment, including a three-dimensional, gadolinium-enhanced magnetization-prepared rapid gradient-echo (MPRAGE) sequence for target volume delineation [26]. All MRIs were obtained on a 3.0T MRI scanner (MAGNETOM Skyra and Prisma, Siemens Healthcare, Erlangen, Germany). The GTV was contoured as the visible tumor on the contrast-enhanced MPRAGE. No additional margin was added to create the clinical target volume (CTV) or PTV for neither mask nor frame immobilization. Treatment doses were prescribed to the highest isodose line (≥50%) encompassing the GTV as per our previously published technique, with no institutional preference to prescribe to a higher isodose line based on the type of immobilization [22]. Plans were optimized for conformity and coverage with a minimum acceptable GTV coverage of ≥99.5%.

### 2.3. Frame Immobilization Workflow

**Figure 1:**
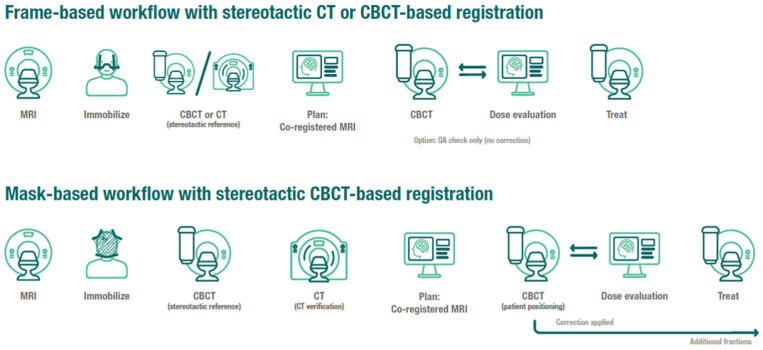
Frame and mask immobilization institutional workflows.

A Leksell stereotactic “G-frame” (Elekta Instrument, AB, Stockholm), a head fixation system with 4 fixation pins, support posts, and a base ring, was affixed to the patient’s head. A mechanical frame adapter was used to attach the frame to the treatment couch. The GK Icon^TM^ platform allows for the acquisition of CBCT, where the imaging isocenter is aligned to the radiation isocenter with an accuracy comparable to frame-based localization and thus can be used for independently defining the stereotactic coordinate space from the frame-defined stereotactic space [27]. Following frame fixation, patients underwent a CBCT (CT dose index (CTDI) 6.3 mGy), which was defined as the stereotactic reference and registered to the planning MRI using a rigid co-registration algorithm. Treatment plans with shots defined in stereotactic space were finalized by the re-optimization/fine-tuning of the pre-planned shots. Prior to treatment, a repeat CBCT (CTDI 2.5 mGy) was performed and co-registered to the stereotactic reference to identify any shifts (Figure 1).

### 2.4. Mask Immobilization Workflow and Treatment Delivery with Motion Management

All patients had individualized thermoplastic masks and cushions (Elekta Icon Mask Nanor, Moldcare headrest) made within 24–48 h of treatment and a stand-alone CBCT (CTDI 6.3 mGy). Treatment planning MRIs were co-registered to the stereotactic CBCT using a mutual information-based co-registration algorithm (Figure 1). A reflective marker was placed on the patient’s nose, which was utilized by the HDMM system to track intrafraction motion continuously during the treatment, and the patient was tracked for at least 5 min to verify immobilization setup and to monitor for patient compliance.

On the day of treatment, patients were set up with the custom headrest, mask, and nose marker. A CBCT (CTDI 2.5 mGy) was obtained and co-registered to the initial reference CBCT to identify any spatial shifts. The resulting registration matrix was used to update shot coordinates, maintaining their location with respect to the anatomy. The dose was calculated using the updated shot coordinates and verified to ensure that all planning metrics were within acceptable deviation (<1%). If an unacceptable deviation occurred, the plan was re-optimized/fine-tuned to meet objectives. The patient was coached during treatment delivery, such that the nose marker was within a maximum excursion of 1.0 mm with respect to the fixed mask fiducials monitored by the HDMM system. Sustained motion > 1.5 mm triggered an automated machine pause, resulting in the cessation of treatment, patient coaching or repositioning as needed, and repeat CBCT with plan evaluation.

### 2.5. Patient Follow-Up and Endpoints

The standard follow-up schedule at our institution after SRS for BM includes multidisciplinary follow-up with radiation oncology and neuro-oncology 8 weeks post-treatment and every 2–3 months subsequently with diagnostic MRI scans and clinical visits. The primary endpoint of this study was freedom from local failure (FFLF) as per Response Assessment in Neuro-Oncology (RANO) criteria for BM [28]. Freedom from distant intracranial failure (DIF) was defined as the time from initial SRS to first development of any new BM outside the previous SRS volumes. Overall survival (OS) was measured as the time from initial SRS to death or last follow-up. Radiation necrosis was defined as new or growing enhancement in the area of prior SRS in which recurrent tumor was excluded. Factors contributing to a diagnosis of radiation necrosis included spontaneous resolution without intracranial anti-tumor therapy, lack of elevated relative cerebral blood volume on dynamic susceptibility contrast MRI perfusion, lack of mass-effect, pathologic confirmation, and/or multidisciplinary tumor conference consensus as per the previously standardized practice [29].

### 2.6. Statistical Analysis

Descriptive statistics were used to describe demographics and clinical characteristics. Categorical variables were reported as frequencies and percentages and compared between the frame and mask immobilization using chi-squared test or Fisher’s exact test. Continuous variables were reported as median and range and compared between the two groups using the Mann–Whitney U test. Kaplan–Meier analysis was used for time-to-event analysis and the log-rank test was used to compare groups. To identify the factors associated with LC, a Cox regression model was used. Variables that showed a significance of *p* ≤ 0.1 in the univariable analysis and immobilization status were included in the multivariable analysis and hazard ratios with 95% confidence intervals (CI) were reported. Statistical significance was set at *p* < 0.05. Statistical analysis was performed using SPSS, version 27 (SPSS Inc., Chicago, IL, USA).

## 3. Results

A total of 150 consecutive patients underwent 189 SRS courses for 453 BM and met the inclusion criteria for this study. Patient and treatment characteristics are presented in Table 1. The median age was 65 (range: 28–90 years), the median KPS was 90 (60–100), and 42.7% were male. The most common primary tumors were lung (55.6%) and breast cancers (18.1%). The prescribed SRS dose was 22 Gy for 107 (23.6%) lesions and 24 Gy for 346 (76.4%) lesions. The mask immobilization cohort included 57 (38%) patients with 129 (23.6%) BM treated in 74 (39.2%) SRS courses, whereas the frame immobilization cohort included 93 (62%) patients who had 115 (60.8%) courses of SRS for 324 (71.5%) BM. The median tumor volume was 0.18 cm^3^ (0.02–2.47 cm^3^) for mask and 0.16 cm^3^ (0.01–3.5 cm^3^) for frame immobilization; the median maximal tumor diameter was 0.8 cm (0.5–1.95 cm) for mask and 0.73 cm (0.5–1.95 cm) for frame immobilization. Frame immobilization treatments were associated with a higher proportion of gastrointestinal cancer metastases and fewer breast cancer metastases (*p* = 0.024), and a higher number of total treated lesions per SRS course (median 7 [1,2,3,4,5,6,7,8,9,10,11,12,13,14,15,16,17,18,19,20,21,22,23] vs. 3 [1,2,3,4,5,6,7,8,9,10,11,12,13,14] lesions; *p* < 0.001). There were no statistically significant differences between the mask and frame immobilization cohorts in terms of age, sex, race, occurrence and status of extracranial disease, KPS, SRS dose, individual tumor volume, or tumor maximal diameter. No differences in any of the several conformity and coverage indices between frame-based and mask-based plans were identified.

**Figure 2:**
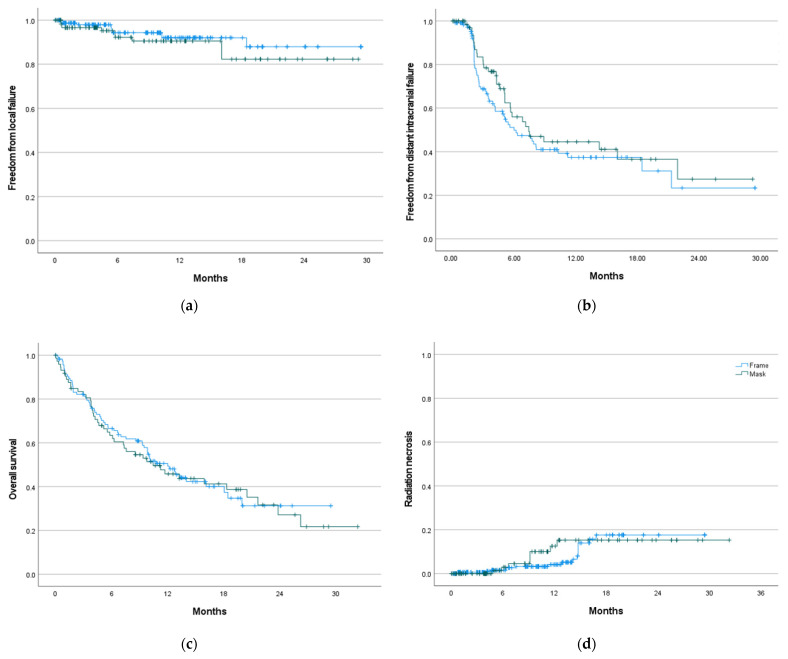
Freedom from local failure (**a**), freedom from distant intracranial failure (**b**), overall survival (**c**), and incidence of radiation necrosis (**d**), by frame- vs. mask-based immobilization with zero-PTV margin.

The median follow-up was 15 months (95% CI: 13–17 months) for the entire cohort; 17.4 months (95% CI: 12.4–22.4 months) for the mask and 15 months (95% CI: 13.5–16.5 months) for the frame immobilization cohorts. On a per lesion basis, 28 (6.2%) local failures (10 failures in mask vs. 18 failures in frame immobilization cohorts) occurred. The median FFLF was not reached. The actuarial FFLF at 6 months and 1 year was 92.2% (95% CI: 86.5–97.6%) and 90.5% (95% CI: 84–97%) for mask, and 94.3% (95% CI: 91.2–97.4%) and 92% (95% CI: 88.1–95.9%) for frame immobilization (*p* = 0.272) (Figure 2a). Median freedom from DIF was 6.8 months (95% CI: 4.9-8.6 months) for the entire cohort; 7.4 months (95% CI: 3.6–11.2 months) with mask and 6.0 months (95% CI: 3.4–8.5 months) with frame immobilization (*p* = 0.312) (Figure 2b). The median OS was 11.2 months (95% CI: 8.8–13.7 months) for all patients; 10.4 months (95% CI: 5.0–15.9 months) for mask and 12 months (95% CI: 9.2-14.9 months) for frame immobilization (*p* = 0.796) (Figure 2c).

There was no statistically significant difference in the incidence of radiation necrosis between the two groups (*p* = 0.502). The cumulative radiation necrosis rate at 6 months and 1 year was 2.9% (95% CI: 2.5–3.3%) and 12.5% (95% CI: 11.6–13.4%) for mask, and 1.5% (95% CI: 1.3–1.7%) and 4.1% (95% CI: 3.8–4.4%) for frame immobilization (Figure 2d).

On both univariable and multivariable analysis, the only factors associated with a shorter time to local failure (all *p* < 0.05) were male (vs. female) sex and presence (vs. absence) of extracranial disease. There was no difference in FFLF between the mask and frame immobilization groups on univariable (*p* = 0.29, HR: 0.81, 95% CI: 0.55–1.19) or multivariable analysis (*p* = 0.518, HR: 0.52, 95% CI: 0.24–1.14) (Table 2). There were no statistically significant differences in the tumor maximum diameter or volume between the lesions that did and did not recur. The median maximal tumor diameter was 0.76 cm (0.5–1.9 cm) for the lesions that recurred and 0.75 cm (0.5–1.95) for the lesions that did not recur (*p* = 0.90). The median tumor volume was 0.15 cm^3^ (0.03–2.53 cm^3^) for the lesions that recurred and 0.17 cm^3^ (0.01–3.46 cm^3^) for the lesions that did not recur (*p* = 0.86).

## 4. Discussion

The optimal SM for treatment of BM with mask immobilization SRS with HDMM on the GK Icon^TM^ has yet to be determined. The study reported in this paper, comprising 150 patients with 453 BM treated in a median of 15 months of follow up, demonstrates that zero-SM mask immobilization had comparable FFLF, DIF, OS, and radiation necrosis compared to a control group of similar patients undergoing frame immobilization SRS. To our knowledge, this analysis represents the largest cohort study comparing zero-SM mask versus frame immobilization on the GK Icon^TM^ to date.

For both mask and frame immobilization SRS on the GK Icon^TM^ system, Duggar et al. reported that the sources of uncertainty include MRI distortion, couch position and stability shifts, CBCT-MRI registration differences, and definition of stereotactic space and intrafraction motion [27]. Uncertainties unique to the mask immobilizations include errors in the definition of stereotactic space using CBCT (0.10 ± 0.05 mm) and MRI-CBCT registration uncertainty (0.62 + 0.23 mm) and is comparable to stereotactic definition with frame immobilization (root-mean-square error smaller than 0.6 to 1.5 mm) as it has been reported [27,30,31]. Carmunicci and colleagues detected an overall average motion error of less than 1 mm in the translational direction and less than or equal to 1° in the rotational direction for both mask and frame immobilization using GK Icon^TM^ [18]. They also showed that the intrafraction error was greater for mask immobilization than for frameimmobilization in all three directions for both translation and rotation. Given the greater risk of motion especially with extended treatment periods, there are some concerns regarding mask immobilization driving the decision to consider the addition of an appropriate SM.

Few studies have reported clinical outcomes comparing mask versus frame immobilization on the GK Icon^TM^. Grimm and colleagues [23] prospectively compared 76 patients with 197 BM treated on the GK Icon^TM^, using either mask (17 patients with 28 BM) or frame immobilization (59 patients with 169 BM). Patients treated with mask immobilization had an additional 1 mm SM, with a minimum 97% PTV coverage by prescription dose (median 22 Gy, range: 16–24 Gy), and 1 mm HDMM threshold. Interestingly, the results demonstrated statistically significant improved LC rates in the mask immobilization group compared to frame immobilization, with no difference in rates of radiation necrosis. The authors cautioned that the results should be interpreted in the context of limited patient numbers, and ultimately concluded that mask immobilization with 1 mm SM does not result in worse LC or radiation necrosis compared to frame immobilization. A study from Wegner and colleagues [21] compared mask and frame immobilization on the GK Icon^TM^ in 95 patients with 374 treated BM. With mask immobilization, the authors used zero-SM for intact BM and 1 mm SM for postoperative cavities. The HDMM threshold was 1 mm and the median prescription dose was 22 Gy (range: 15–24 Gy). After propensity score matching and a median follow up of 5 months, 10 lesions had a local failure, resulting in a 1-year LC of 85% with mask versus 96% with frame immobilization (*p* = 0.07). On multivariable analysis, mask versus frame immobilization was not associated with local failure. The authors concluded that mask immobilization resulted in comparable outcomes with frame immobilization with short-term follow up, similar to our larger study with longer follow up. Although the incidence of radiation necrosis in our study was numerically higher in the mask immobilization group at 1 year, by 16 months the curves converge and overall there was no statistically significant difference in radiation necrosis rates. However, because many patients were censored prior to this time point, longer follow up and additional patients are required to adequately assess a potential differential radiation necrosis risk. A summary of our results and the aforementioned studies are displayed in Table 3.

Our study has several strengths. First, this study had larger numbers of patients and lesions (150 patients with 453 BM) with a relatively longer follow-up time than previous GK Icon^TM^ mask and frame immobilization comparison studies [21,23]. Second, we used strict inclusion criteria, including only lesions 0.5–2.0 cm in maximal diameter treated to 22–24 Gy, and excluded cases of fractionated SRS, postoperative cavities, and patients who received prior WBRT, which made our cohort more homogenous. Limitations include those inherent to a retrospective study and the potential over-estimation of local failure with the statistical methods used. Patients with a higher number of lesions were preferentially treated with frame immobilization because of the expected longer treatment times. However, this was accounted for in the multivariable analysis. Although motion with mask immobilization may be greatest among peripheral lesions, as opposed to those located centrally within the brain, we did not collect or analyze data based on distance from the center of the brain. Subsequent analyses to explore this variable are warranted and currently under development. However, there was no institutional preference on the type of immobilization based on central versus peripheral location of lesions. We also excluded all lesions smaller than 0.5 cm in maximal diameter since these were typically treated with frame immobilization per institutional preference, limiting extrapolation of these results to punctate lesions. Furthermore, our institutional prescription dose for BM ≤ 2.0 cm in maximal diameter is 22–24 Gy with ≥99.5% coverage, both of which may be higher than those used by other institutions for a similar cohort. This may account for high LC rates even with sub-millimeter positioning variances. With a higher prescription dose and target coverage requirement, there is a resultant small unintentional dosimetric margin extending beyond the target, which may result in a similar dosimetry to that of a plan with a lower prescription dose with a small SM added to generate the PTV. Lastly, longer-term follow-up is required to better assess outcomes.

## 5. Conclusions

Mask immobilization with zero-SM using CBCT for interfraction localization and HDMM for intrafraction monitoring on the GK Icon^TM^ produces comparable outcomes compared to traditional frame immobilization for BM of 0.5–2.0 cm in maximal diameter treated with single fraction SRS using 22–24 Gy. A longer follow-up is required, but the initial findings support omitting a SM, and thus PTV margin, when using mask immobilization with this overall treatment approach.

## Figures and Tables

**Table 1:** Patient, tumor, and treatment characteristics of the total, frame, and mask immobilization cohorts.

	Total Cohort (% or Range)	Frame Immobilization Cohort (% or Range)	Mask Immobilization Cohort (% or Range)	*p*-Value
Number of patients	150	93	57	
Median age, years	65 (28–90)	65 (28–90)	66 (28–89)	0.876
Sex				0.398
Female	86 (57.3)	56 (60.2)	30 (52.6)	
Male	64 (42.7)	37 (39.8)	27 (47.4)	
Race				0.052
White	137 (91.3)	89 (95.7)	48 (84.2)	
African American	10 (6.7)	3 (3.2)	7 (12.3)	
Other	3 (2.0)	1 (1.1)	2 (4.5)	
Extracranial disease				0.113
No	109 (24.1)	71 (21.9)	38 (29.4)	
Yes	344 (75.9)	253 (78.1)	91 (80.6)	
Status of extracranial disease				0.143
Stable	201 (44.4)	151 (46.6)	50 (38.8)	
Progressive	252 (55.6)	173 (53.4)	79 (61.2)	
Median KPS	90 (60–100)	90 (60–100)	90 (60–100)	0.184
Primary tumor histology				0.024
Lung	252 (55.6)	176 (54.3)	76 (58.9)	
Breast	82 (18.1)	54 (16.7)	28 (21.7)	
Gastrointestinal	43 (9.5)	39 (12.0)	4 (3.1)	
Other	76 (16.8)	55 (16.9)	21 (16.3)	
Number of SRS courses	189	115	74	
Number of brain metastases	453	324	129	
Median number of brain metastases treated per SRS course	6 (1–23)	7 (1–23)	3 (1–14)	<0.001
SRS prescription dose				0.086
22 Gy	107 (23.6)	84 (25.9)	23 (17.8)	
24 Gy	346 (76.4)	240 (74.1)	106 (82.2)	
Prescription Isodose Line (%)	56 (50–94)	56 (50–94)	55 (50–94)	0.860
Median maximal tumor diameter, cm	0.8 (0.5–1.95)	0.73 (0.5–1.95)	0.8 (0.5–1.95)	0.068
Median tumor volume, cm^3^	0.18 (0.01–3.5)	0.16 (0.01–3.5)	0.18 (0.02–2.47)	0.054

**Table 2:** Univariable and multivariable analyses for local failure.

	Univariable Analysis		Multivariable Analysis	
Variables	HR (95% CI)	*p*-Value	HR (95% CI)	*p*-Value
Age	1.02 (0.99, 1.04)	0.156		
Sex		0.022		0.008
Male	Reference		Reference	
Female	0.64 (0.43, 0.94)		0.35 (0.16, 0.76)	
Primary tumor histology		0.411		
Other	Reference			
Lung	1.60 (0.84, 3.04)			
Breast	1.28 (0.53, 3.14)			
Gastroinestinal	0.81 (0.26, 2.50)			
Total number of brain metastases treated per SRS course	0.97 (0.90, 1.04)	0.374		
Extracranial disease		0.006		0.016
Yes	Reference		Reference	
No	0.45 (0.22, 0.92)		0.17 (0.40, 0.72)	
Status of extracranial disease		0.251		
Stable	Reference			
Progressive	1.24 (0.86, 1.80)			
KPS	1.01 (0.97, 1.05)	0.773		
Immobilization method		0.290		0.518
Mask	Reference		Reference	
Frame	0.81 (0.55, 1.19)		0.52 (0.24, 1.14)	
SRS prescription dose		0.336		
24 Gy	Reference			
22 Gy	1.26 (0.77, 2.04)			
Tumor maximal diameter	1.33 (0.52, 3.38)	0.561		
Tumor volume	1.16 (0.63, 2.14)	0.636		

**Table 3:** Overview summary of Gamma Knife^®^ Icon^TM^ studies.

	Patient Number	Lesion Number	Median SRS Dose	Setup Margin	HDMM Threshold	Median Follow-Up	Local Control Rate	Overall Survival Rate	Radiation Necrosis Rate
Grimm et al. [23]	Mask:17 Frame: 59	Mask: 28 Frame: 169	22 Gy (10 patients FSRT, 24 patients had prior WBRT)	Mask: 1 mm Frame: Zero	1 mm	Mask: 10.4 months Frame: 9 months	Mask: 6-month and 1-year 100% Frame: 6-month 92.6%, 1-year 80.9% (*p* = 0.03)	Mask: not reached Frame: 16.9 months (*p* = 0.999)	Mask: Zero event Frame: 3 events (*p* = 0.67)
Wegner et al. [21]	Mask: 56 Frame: 39	Mask: 80 Frame: 80 (after propensity matching)	20 Gy (20 patients had prior WBRT)	Mask: Zero for intact metastases, 1 mm for postoperative cavities Frame: Zero for intact metastases, 1 mm for postoperative cavities	1 mm	Entire cohort: 5 months clinical follow-up 6 months imaging follow-up	Mask: 1-year 85% Frame: 1-year 96% (*p* = 0.07)	Mask: not reached, 1-year 75% Frame: 8 months, 1-year: 48% (*p* = 0.12)	NA
Kutuk et al.	Mask: 57 Frame: 93	Mask: 129 Frame: 324	24 Gy	Mask: Zero Frame: Zero	1.5 mm	Mask: 17.4 months Frame: 15 months	Mask: 6-month 92.2% and 1-year 90.5% Frame: 6-month 94.3%, 1-year 92% (*p* = 0.272)	Mask: 10.4 months Frame: 12 months (*p* = 0.796)	Mask: 6-month 2.9% and 1-year 12.5% Frame: 6-month 1.5% and 1-year 4.1% (*p* = 0.502)

## Data Availability

Research data are stored in an institutional repository and will be shared upon request to the corresponding author.
